# Digitalisierungsschub in Firmen während der Corona-Pandemie

**DOI:** 10.1007/s10273-021-3005-3

**Published:** 2021-09-21

**Authors:** Lutz Bellmann, Pauline Bourgeon, Christina Gathmann, Christian Kagerl, David Marguerit, Ludivine Martin, Laura Pohlan, Duncan Roth

**Affiliations:** 1 Forschungsbereich Betriebe und Beschäftigung, Institut für Arbeitsmarkt- und Berufsforschung (IAB), Regensburger Straße 104, 90478 Nürnberg, Deutschland; 2 Labour Market Department , Luxembourg Institute of Socio-Economic Research (LISER), 11, Porte des Sciences , L-4366 Esch-sur-Alzette/Belval, Luxemburg; 3 Alfred-Weber-Institut für Wirtschaftswissenschaften, Ruprecht-Karls-Universität Heidelberg, Bergheimer Straße 20, 69115 Heidelberg, Deutschland; 4 Institut für Arbeitsmarkt- und Berufsforschung (IAB), Regensburger Straße 104, 90478 Nürnberg, Deutschland; 5 Luxembourg Institute of Socio-Economic Research (LISER), 11, Porte des Sciences , L-4366 Esch-sur-Alzette/Belval, Luxemburg; 6 Arbeitsmarktprozesse und Institutionen, Institut für Arbeitsmarkt- und Berufsforschung (IAB), Regensburger Straße 104, 90478 Nürnberg, Deutschland; 7 Regionales Forschungsnetz, Institut für Arbeitsmarkt- und Berufsforschung (IAB), Regensburger Straße 104, 90478 Nürnberg, Deutschland

**Keywords:** J24, L23, I18

## Abstract

Durch die Corona-Pandemie haben digitale Technologien in Unternehmen an Bedeutung gewonnen. Auf Basis einer Betriebsbefragung des Instituts für Arbeitsmarkt- und Berufsforschung untersuchen die Autor:innen, ob Unternehmen vermehrt in digitale Technologien investiert haben und welche Rolle dabei die wirtschaftliche Situation gespielt hat. Neben den Investitionen in digitale Technologien haben auch Weiterbildungsaktivitäten in den Betrieben zugenommen.

## Abstract (en)

This article shows the extent to which companies’ investments in digital technologies have increased during the COVID-19 pandemic. Based on a large German company survey conducted by the Institute for Employment Research, the authors examine whether and which companies have invested in different types of digital technologies, how these investments are connected to working from home, and how investments are influenced by the economic situation of companies during the pandemic. The authors also discuss further training activities provided and planned by employers and how those are related to their investments in digital technologies.

Die COVID-19-Pandemie hat durch Unterbrechungen der Lieferketten, Verzögerungen in den Produktionsabläufen und eine einbrechende oder sich verschiebende Nachfrage erhebliche Auswirkungen auf die Wirtschaft. Gleichzeitig mussten Firmen ihre Produktions- und Arbeitsprozesse an die infektiöse Dynamik und die Maßnahmen zu deren Eindämmung anpassen. Die massive Ausweitung von Homeoffice hat z.B. zu dramatischen Änderungen geführt, wo und wie Beschäftigte ihre Arbeit verrichten (Taneja et al., 2021). Auch Betriebe in Deutschland haben im Zuge der Pandemie ihren Beschäftigten vermehrt die Option auf Homeoffice gewährt (Bellmann et al., 2020). In vielen Organisationen und Tätigkeiten kommt daher digitalen Technologien für die Kommunikation zwischen Mitarbeitenden, der Organisation von Arbeitsabläufen und der Sicherung von Firmendaten eine zentrale Rolle zu. Während die Unsicherheit über die Zukunft Investitionen in neue Technologien eher verhindert oder zumindest verzögert haben könnte, sollte die Notwendigkeit, persönliche Kontakte zu reduzieren und Arbeitsunterbrechungen zu bewältigen, Investitionen in digitale Technologien tendenziell erhöht haben.

Zwar gibt es Hinweise, dass es durch die Pandemie zu einer beschleunigten Digitalisierung gekommen ist (OECD, 2021) und dass auch in Deutschland ein Digitalisierungsschub stattgefunden hat (Bertscheck, 2020). Gleichzeitig sind Untersuchungen auf der Grundlage von Unternehmensdaten, die Erkenntnisse dazu liefern könnten, wie Firmen auf die doppelte Herausforderung der steigenden Unsicherheiten und Bedürfnisse reagierten, selten.

Unsere Ergebnisse basieren auf einer großen Betriebsbefragung „Betriebe in der COVID-19-Krise" (Aminian et al., 2021), die das Institut für Arbeitsmarkt- und Berufsforschung (IAB) seit August 2020 durchführt. Die Daten der neunten Welle umfassen 1.941 Betriebe in ganz Deutschland, die im Februar 2021 telefonisch befragt wurden. Die Ergebnisse sind gewichtet, um repräsentativ für alle privaten Betriebe mit mindestens einem sozialversicherungspflichtig Beschäftigten zu sein. Deutschland stellt einen besonders interessanten Fall dar, da die digitale Infrastruktur in vielen Bereichen hinterherhinkt (Wissenschaftlicher Beirat des BMWi, 2021).

## Haben Firmen in digitale Technologien investiert?

Seit Beginn der Pandemie hat fast die Hälfte aller Betriebe in digitale Technologien wie Hardware, Software oder digitale Infrastruktur investiert (vgl. Abbildung 1). Größere Betriebe investieren dabei häufiger in digitale Technologien als kleinere. Unter den Betrieben mit 250 und mehr Beschäftigten hat fast jeder Betrieb Investitionen getätigt, aber nur 39 % der Betriebe mit weniger als zehn Beschäftigten. Am häufigsten wurde im Informations- und Kommunikationssektor (57 %) und bei sonstigen Dienstleistungen[1] (58 %), seltener im verarbeitenden Gewerbe (34 %), im Baugewerbe (36 %) und im Handel (39 %) investiert.

Auch ohne die Pandemie hätten die Betriebe möglicherweise in digitale Technologien investiert. Hat die Pandemie also tatsächlich die Verbreitung digitaler Technologien in den Betrieben beschleunigt? Fast 30 % der Betriebe ge-

1 Hierzu gehören „Erbringung von freiberuflichen, wissenschaftlichen und technischen Dienstleistungen", „Erbringung von sonstigen wirtschaftlichen Dienstleistungen", „Erbringung von Finanz- und Versicherungsdienstleistungen" und „Kunst und Unterhaltung".

**Prof. Dr. Lutz Bellmann** leitet den Forschungsbereich Betriebe und Beschäftigung des Instituts für Arbeitsmarkt- und Berufsforschung (IAB) in Nürnberg und hat den Lehrstuhl für Volkswirtschaftslehre, insb. Arbeitsökonomie, an der Friedrich-Alexander-Universität Erlangen-Nürnberg inne.

**Pauline Bourgeon, Ph. D.**, ist Researcher, **David Marguerit** ist Policy Analyst und **Ludivine Martin** ist Research Scientist am Luxembourg Institute of Socio-Economic Research (LISER).

**Prof. Christina Gathmann, Ph. D.**, ist Leiterin der Arbeitsmarktabteilung am Luxembourg Institute of Socio-Economic Research (LISER) und Professorin an der Universität Luxemburg.

**Christian Kagerl** ist wissenschaftlicher Mitarbeiter in der Forschungsgruppe des Direktors am IAB.

**Dr. Laura Pohlan** ist wissenschaftliche Mitarbeiterin im IAB-Forschungsbereich „Arbeitsmarktprozesse und Institutionen" und in der ZEW-Forschungsgruppe „Marktdesign".

**Dr. Duncan Roth** ist Leiter der Nachwuchsforschungsgruppe Berufe und Erwerbsverläufe am IAB.

ben an, dass die Krise die Einführung oder den Ausbau digitaler Technologien in ihrem Betrieb beschleunigt hat (vgl. Abbildung 2). Gleichzeitig berichten 69 %, dass sich die Investitionen nicht verändert haben, während 2 % der Betriebe sagen, dass die Krise die Verbreitung digitaler Technologien verlangsamt hat.

Hinter den Gesamtzahlen verbergen sich wiederum große sektorale Unterschiede. Betriebe in den Sektoren Information und Kommunikation (43 %), Bildung, Gesundheit und Soziales (31 %) sowie sonstige Dienstleistungen (41 %) geben am ehesten an, Investitionen in digitale Technologien aufgrund der Pandemie erhöht zu haben. Im Baugewerbe sowie im Sektor Verkehr und Lagerei

Abbildung 1
**Investitionen in IT oder digitale Technologien seit Beginn der COVID-19-Pandemie**

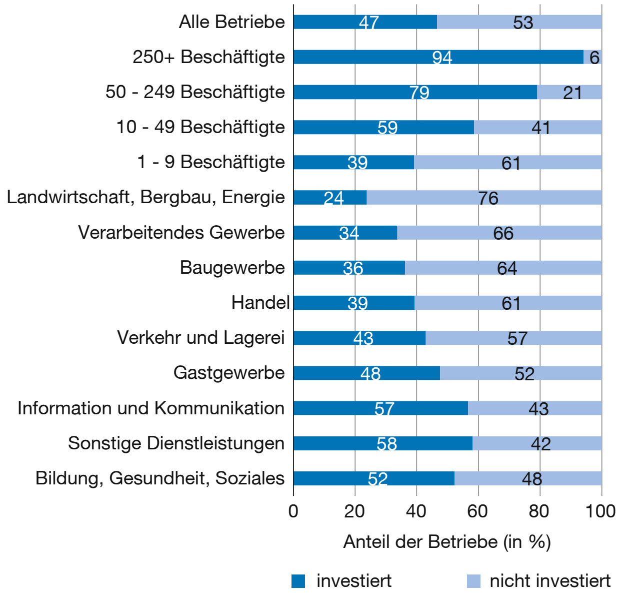

Anmerkungen: Die Abbildung zeigt gewichtete Anteile auf Basis von 1.941 befragten Betrieben im Februar 2021.

Quelle: eigene Berechnungen.

– und damit in Sektoren, in denen das Arbeiten von zuhause schwierig ist – sagen nur 12 % bzw. 17 % der Betriebe, dass die Pandemie die Investitionen in digitale Technologien beschleunigt hat. Im Gastgewerbe, einem Sektor, der stark von den Nachfrageausfällen während der Schließungen betroffen war, sagen 10 % der Betriebe, dass die Krise sogar die Geschwindigkeit der Verbreitung digitaler Technologien verringert hat. Auch nach Betriebsgröße gibt es eine deutliche Kluft: Fast 75 % der großen Betriebe (250+ Beschäftigte) sehen die Pandemie als Beschleuniger der Digitalisierung, während nur etwa ein Viertel der kleinen (weniger als zehn Beschäftigte) und ein Drittel der mittelgroßen (zehn bis 49 Beschäftigte) Betriebe diese Ansicht teilen.

Die meisten Betriebe, die nicht investiert haben, sagen, dass solche Investitionen nicht als notwendig erachtet wurden. Nur 15 % der Betriebe geben an, dass die Investitionen zu teuer gewesen wären und 12 % berichten, dass die Planung und Umsetzung zu komplex gewesen wäre. Solche Investitionshemmnisse sind für kleine und mittlere Betriebe, die eher unter Liquiditätsengpässen leiden, viel relevanter. Hohe Kosten wurden als wichtiges Investitionshindernis im Handel und im Gastgewerbe angesehen, zwei Sektoren, die von der Krise und den Maßnahmen zu ihrer Eindämmung besonders betroffen waren.

Abbildung 2
**Die COVID-19-Pandemie und die Verbreitung digitaler Technologien**

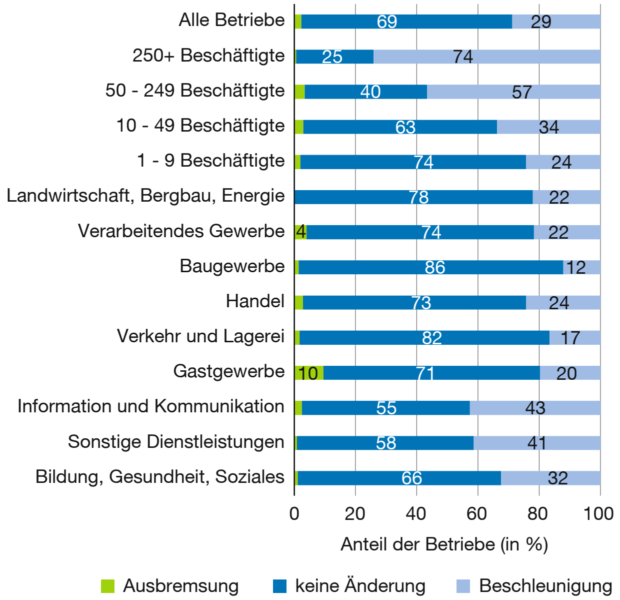

Anmerkungen: Die Abbildung zeigt gewichtete Anteile auf Basis von 1.941 befragten Betrieben im Februar 2021.

Quelle: eigene Berechnungen.

## In welche Art von digitalen Technologien haben Firmen investiert?

Die Betriebe werden auch nach der Art der digitalen Technologie gefragt, in die investiert wurde. Dabei wird zwischen Hardware (z. B. Laptops, Smartphones) und Software zur gemeinsamen Dokumentenverwaltung und Zusammenarbeit (z. B. SharePoint, Google Doc) oder Software zur digitalen Kommunikation und Prozessautomatisierung (z. B. Microsoft Teams, Zoom) unterschieden. Weitere Investitionskategorien sind der Fernzugriff, damit Mitarbeitende Datenbanken und Dateien sicher nutzen können (z. B. VPN-Verbindung), Internetgeschwindigkeit, Datenschutz und IT-Sicherheit und schließlich zusätzliches IT-Personal.

Betriebe investierten am häufigsten in Hardware (vgl. Abbildung 3). 41 % aller Betriebe kauften Computer, Laptops, Smartphones oder Headsets. Investitionen in Software sind ebenfalls häufig, vor allem in Kommunikations- und Prozessautomatisierungssoftware (23 %), gefolgt von Investitionen in Software für die Zusammenarbeit (19 %). Investitionen in schnelleres Internet (9 %) oder zusätzliches IT-Personal (2 %) waren deutlich weniger wichtig.

Nicht alle diese Investitionen wurden jedoch aufgrund der Pandemie getätigt. Abbildung 3 zeigt, dass die Krise vor allem Investitionen in Kommunikationssoftware beflügelt

Abbildung 3
**Art der Investitionen in IT oder digitale Technologien**

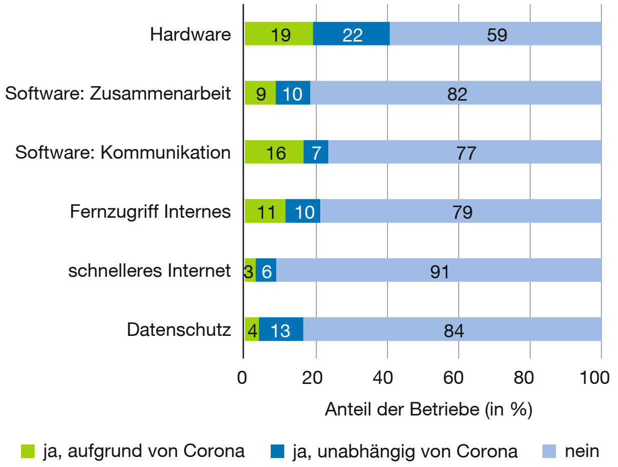

Anmerkungen: Die Abbildung zeigt gewichtete Anteile auf Basis von 1.941 befragten Betrieben im Februar 2021.

Quelle: eigene Berechnungen.

hat. Kommunikationstools wie Microsoft Teams oder Zoom sind wichtige digitale Werkzeuge, um Arbeitsprozesse effektiv zu koordinieren und zu kommunizieren, wenn die Beschäftigten räumlich getrennt sind. Sie sind eine wichtige Grundvoraussetzung oder Ergänzung zur Ausweitung des Homeoffice. Darüber hinaus hat fast die Hälfte der Betriebe, die in Hardware, in Software für die Zusammenarbeit und in Fernzugriff investiert haben, dies wegen der Pandemie getan. Für Investitionen in Internetgeschwindigkeit, IT-Personal oder Datenschutz hat die Pandemie dagegen eine weniger prominente Rolle gespielt.

Interessanterweise sind es vor allem Betriebe im Dienstleistungssektor, die ihre Investitionen in alle Arten von digitalen Technologien deutlich erhöht haben. Betriebe im Bildungs-, Gesundheits- und Sozialwesen investierten vor allem in Hardware und Kommunikationssoftware, während Betriebe im Bereich Information und Kommunikation vor allem in Software für die Zusammenarbeit investierten. Auch hier gilt, dass COVID-19-bedingte Investitionen in IT und Digitalisierung in größeren Betrieben deutlich wahrscheinlicher sind als in kleineren Betrieben, insbesondere in Bezug auf Hardware und Fernzugriff.

## Wie haben sich die betrieblichen Bedingungen auf die Investitionen ausgewirkt?

Die erheblichen Unterschiede in den Investitionstätigkeiten zwischen den einzelnen Sektoren könnten darauf hinweisen, dass die betrieblichen Bedingungen, mit denen die Betriebe in ihrem Tätigkeitsbereich konfrontiert waren, Investitionen in neue Technologien gefördert oder gehemmt haben könn-

Abbildung 4

**Investitionen in IT und digitale Technologien nach betrieblichen Bedingungen**

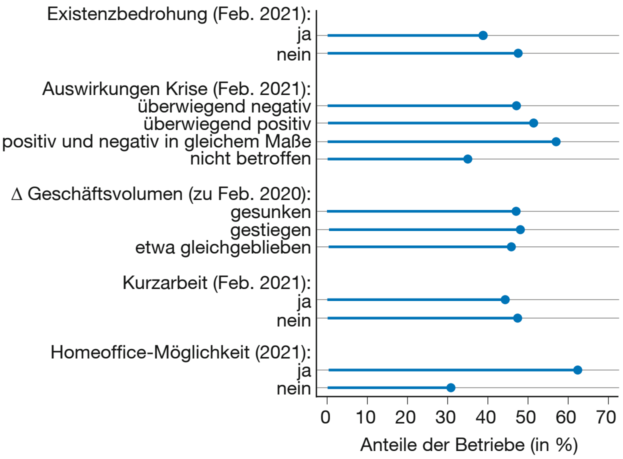

Anmerkungen: Die Abbildung zeigt gewichtete Anteile auf Basis von 1.941 Betrieben, die im Februar 2021 befragt wurden. Die Information, ob Homeoffice möglich ist, stammt aus den Befragungswellen im Januar, März und April 2021 und ist für 1.506 Betriebe verfügbar.

Quelle: eigene Berechnungen.

ten. In der Tat zeigt Abbildung 4, dass die wirtschaftlichen Schwierigkeiten, die Unternehmen während der Pandemie erlebten, ihre Spuren hinterlassen haben: Betriebe, die im Februar 2021 am Rande der Insolvenz stehen, investieren seltener als Betriebe, die nicht um ihre Existenz besorgt sind; allerdings ist der Unterschied (9 %) geringer, als man erwarten würde. Auch Betriebe, die im Februar 2021 überwiegend negativ von der Krise betroffen sind, investieren mit geringerer Wahrscheinlichkeit als Betriebe, die positiv betroffen sind oder sowohl positive als auch negative Auswirkungen erfahren haben. Interessant ist, dass Betriebe am wenigsten in neue Technologien investieren, wenn ihr Geschäft nicht von der Pandemie betroffen war. Der Schock einer globalen Pandemie und die damit verbundene wirtschaftliche Anfälligkeit beschleunigten also die Entscheidung der Betriebe, ihre digitale Infrastruktur zu aktualisieren. Ein wichtiger Treiber für Investitionen in digitale Technologien war dabei die Möglichkeit, Beschäftigte von zuhause arbeiten zu lassen. 62 % der Betriebe, die Homeoffice ermöglichten, investierten in digitale Technologien, aber nur 31 % der Betriebe, die keine Möglichkeit für Homeoffice sahen.

Dagegen scheint die tatsächliche wirtschaftliche Lage eines Betriebs für Investitionsentscheidungen weniger wichtig zu sein. Betriebe, die Kurzarbeit nutzen, um einen Rückgang in der Produktion oder Dienstleistungserbringung auszugleichen, investieren mit gleicher Wahrscheinlichkeit wie Betriebe ohne Kurzarbeit. Ebenso ist die Entscheidung, in digitale Technologien zu investieren, in Betrieben mit Umsatzrückgängen und Betrieben mit Umsatzsteigerungen im letzten Jahr ähnlich. Diese Muster deuten darauf hin, dass die Betriebe mehr auf den Schock einer globalen Pandemie reagiert haben, aber weniger auf die aktuelle wirtschaftliche Situation, die viele wahrscheinlich als vorübergehend betrachtet haben oder dies zumindest hofften.

## Betriebe mit Investitionen in digitale Technologien führen auch häufiger IT-Schulungen durch

Investitionen in digitale Technologien könnten ebenso einen gesteigerten Bedarf an Schulungen im Umgang mit diesen Technologien unter den Mitarbeitenden mit sich bringen (Janssen et al., 2018). Etwa jeder fünfte Betrieb gibt an, seit Beginn der Pandemie IT-Schulungen durchgeführt zu haben und knapp 40 % berichten, dass solche Schulungen 2021 und 2022 geplant sind. Dass es sich hierbei nicht bloß um das übliche Schulungsgeschehen handelt, zeigt sich auch darin, dass mit 45 % fast die Hälfte der Betriebe den Schulungsumfang aufgrund der Pandemie erhöht hat.

Wie auch bei den Investitionen in digitale Technologien stellt sich die Durchführung von IT-Schulungen nicht einheitlich dar, sondern fällt für Betriebe sehr unterschiedlich aus. Mit Blick auf die Betriebsgröße zeigt sich erwartungsgemäß, dass IT-Schulungen mit zunehmender Betriebsgröße häufiger durchgeführt worden sind und auch in Zukunft öfter geplant sind. So haben Schulungen in 62 % der Großbetriebe stattgefunden, und in 88 % sind sie für 2021 und 2022 geplant, während die entsprechenden Anteile in Kleinstbetrieben lediglich bei 17 % bzw. 33 % liegen. Interessanterweise fällt die pandemiebedingte Veränderung im Umfang der IT-Schulungen jedoch bei Groß- und Kleinstbetrieben nahezu identisch aus – in beiden Fällen berichtet etwa die Hälfte der Betriebe von einem Anstieg der Schulungen.

Besonders häufig haben IT-Schulungen in Betrieben des Wirtschaftszweigs Information und Kommunikation (41 %), im Bildungs-, Gesundheits- und Sozialwesen (30 %) sowie in den sonstigen Dienstleistungen (29 %) stattgefunden und somit in den Wirtschaftszweigen, in denen auch Investitionen in digitale Technologien häufiger vorgenommen wurden. Dieselben Wirtschaftszweige sind auch bei den geplanten Schulungen am stärksten vertreten. Dagegen fällt der pandemiebedingte Anstieg im Bereich Information und Kommunikation mit 31 % nur unterdurchschnittlich aus, was auf ein allgemein hohes Schulungsniveau hindeutet, das im Zuge der COVID-19-Krise weniger häufig angepasst werden musste. Anders verhält es sich bei Betrieben im Sektor Verkehr und Lagerei, in denen 70 % von einem pandemiebedingten Anstieg berichten.

Der positive Zusammenhang zwischen Investitionen in digitale Technologien und IT-Schulungen zeigt sich auch auf der Betriebsebene. In Betrieben, die in digitale Technologien investiert haben, haben Schulungen in 37 % der Fälle stattgefunden und damit deutlich häufiger als in Betrieben ohne Investitionen (9 %). Entsprechend höher fällt auch der Anteil der Betriebe aus, in denen Schulungen in Zukunft geplant sind (59 % gegenüber 25 %). Betriebe, die pandemiebedingt in digitale Technologien investiert haben, haben deutlich häufiger auch IT-Schulungen angeboten (in 45 % der Fälle) als Betriebe, die aus anderen Gründen in digitale Technologien investiert haben (nur 25 %).

Ergebnisse einer multivariaten Untersuchung, in denen für die Größe und den Wirtschaftszweig eines Betriebs kontrolliert wird, bestätigen den Befund, dass Investitionen in digitale Technologien mit einem höheren Schulungsumfang einhergehen. Dabei zeigt sich, dass Betriebe, die aufgrund der COVID-19-Krise in digitale Technologien investiert haben, eine im Durchschnitt um 29 Prozentpunkte höhere Wahrscheinlichkeit aufweisen, IT-Schulungen durchzuführen, als Betriebe, die nicht investiert haben. Dagegen weisen Betriebe, die unabhängig von der Pandemie Investitionen getätigt haben, lediglich eine um 15 Prozentpunkte höhere Schulungswahrscheinlichkeit auf. Somit hat die Corona-Pandemie nicht nur Investitionen in digitale Technologien beschleunigt, sondern ebenso zu einer deutlichen Ausweitung von IT-Schulungen durch die Arbeitgebenden geführt.

## Weiterbildungsbedarf in der Kommunikations- und Kooperationsfähigkeit sowie beim Datenschutz

Hat die Ausweitung der IT-Schulungen den zusätzlichen Bedarf an Weiterbildung decken können? Die Daten der Betriebsbefragung legen nahe, dass es durch die pandemiebedingten Umstellungen in den betrieblichen Arbeitsabläufen einen gesteigerten Bedarf nach Weiterbildung in verschiedenen Kompetenzbereichen gibt. Wie in Abbildung 5 zu sehen, geben etwa 27 % der Betriebe immer noch einen Weiterbildungsbedarf bezüglich Kommunikations- und Kooperationsfähigkeiten an, was darauf zurückzuführen sein kann, dass im Zuge der erweiterten Homeoffice-Möglichkeiten bisherige Kommunikationsprozesse angepasst werden mussten. Jeweils knapp 37 % der Betriebe sehen dagegen keinen Weiterbildungsbedarf in diesem Bereich bzw. schätzen diese Kompetenz als nicht relevant ein. Der Datenschutz wird von 22 % der Betriebe als relevantes Qualifikationsfeld angegeben, während 52 % bzw. 26 % der Betriebe keinen Weiterbildungsbedarf sehen bzw. die Kompetenz als nicht relevant einschätzen. Mit Anteilen zwischen 12 % und 19 % fällt die Notwendigkeit zur Weiterqualifikation in den Bereichen IT-Anwendungen, IT-Fachwissen und Softwareprogrammierung, Führungskompetenzen sowie Selbstständigkeit, Planungssicherheit und Organisationsfähigkeit geringer aus, wobei mehr als die Hälfte der Betriebe die Kompetenz IT-Fachwissen und Softwareprogrammierung als für sie nicht relevant einstuft.

Abbildung 5
**Weiterbildungsbedarfe in Betrieben in Deutschland**

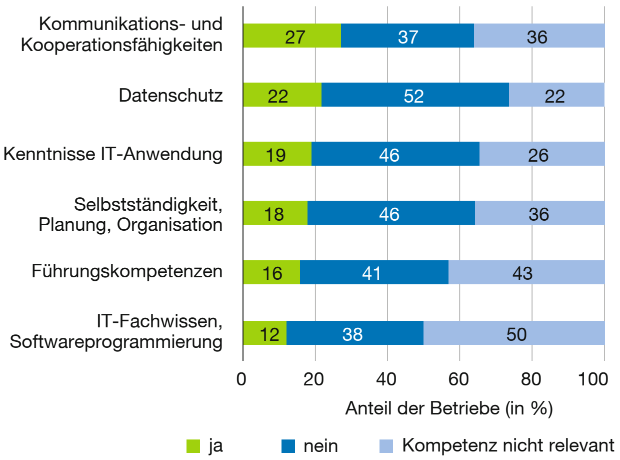

Anmerkungen: Die Abbildung zeigt gewichtete Anteile auf Basis von 1.941 befragten Betrieben im Februar 2021.

Quelle: eigene Berechnungen.

Auch mit Blick auf die Weiterbildungsbedarfe variieren die Ergebnisse mit verschiedenen betrieblichen Merkmalen. Im Durchschnitt schätzen größere Betriebe den Weiterbildungsbedarf in allen Kompetenzfeldern höher ein, während es vor allem kleinere Betriebe sind, die eine bestimmte Kompetenz als nicht relevant betrachten. Z. B. geben 65 % der Großbetriebe einen Bedarf nach Qualifikation im Kompetenzfeld Kommunikation und Kooperationsfähigkeit an, aber nur 23 % der Kleinstbetriebe. Im Fall des Datenschutzes liegen die entsprechenden Anteile bei 52 % bzw. 20 %.

Vergleicht man die verschiedenen Wirtschaftszweige, ist in den Bereichen Information und Kommunikation sowie bei den sonstigen Dienstleistungen für jede Kompetenz der Anteil der Betriebe unterdurchschnittlich, die diesen Bereich als nicht relevant betrachten. Demgegenüber fällt nur für die sonstigen Dienstleistungen der Anteil der Betriebe, der einen Weiterbildungsbedarf angibt, für jedes Kompetenzfeld überdurchschnittlich aus.

Bei Betrieben, die in digitale Technologien investiert haben, fällt der Anteil mit Weiterbildungsbedarf in allen Kompetenzfeldern höher aus als bei Betrieben ohne solche Investitionen. Besonders ausgeprägt ist dieser Unterschied im Fall des Weiterbildungsbedarfs in den Bereichen Führungskompetenzen sowie Kommunikations- und Kooperationsfähigkeiten. Berücksichtigt man darüber hinaus, ob Betriebe pandemiebedingt oder aus anderen Gründen investiert haben, zeigt sich, dass der höhere Weiterbildungsbedarf unter investierenden Betrieben vor allem auf solche mit pandemiebedingten Investitionen zurückzuführen ist. Unter Berücksichtigung von Größe und Wirtschaftszweig eines Betriebs fällt die durchschnittliche Wahrscheinlichkeit für Betriebe mit pandemiebedingten Investitionen in digitale Technologien im Fall der Kommunikations- und Kooperationsfähigkeit um 29 Prozentpunkte, im Fall des Datenschutzes um 21 Prozentpunkte höher aus als bei Betrieben ohne Investitionen. Wie im Fall der IT-Schulungen deuten diese Ergebnisse also darauf hin, dass es insbesondere Betriebe mit pandemiebedingten Investitionen in digitale Technologien sind, die einen höheren Weiterbildungsbedarf sehen.

## Diskussion

In Deutschland haben etwa 50 % aller Betriebe seit Beginn der Corona-Krise in irgendeine Form von digitalen Technologien investiert; und 30 % aller Betriebe betrachten die Pandemie als Beschleunigerin der Digitalisierung. Besonders ausgeprägt sind die Investitionen in Großbetrieben, während kleine und mittlere Betriebe seltener investieren und eher mit finanziellen oder logistischen Barrieren zu kämpfen haben. Hardware stellt die häufigste Art digitaler Investitionen dar, während Investitionen in Kommunikationssoftware durch die Pandemie am meisten beschleunigt wurden.

Die Möglichkeit, Beschäftigte im Homeoffice arbeiten zu lassen, ist ein wichtiger Treiber für die Erneuerung der digitalen Infrastruktur. Doch auch Betriebe, in denen Homeoffice keine Option war, investierten in digitale Technologien. Für die Investitionsentscheidung scheint die Erfahrung einer globalen Pandemie, die in manchen Bereichen der Wirtschaft einen erheblichen Nachfragerückgang verursachte sowie Unterbrechungen in den Produktionsabläufen nach sich zog und dadurch mehrere wirtschaftliche Schwachstellen aufdeckte, wichtiger gewesen zu sein als die aktuelle wirtschaftliche Situation eines Betriebs. Für die Zukunft wird hierbei interessant sein, wie sich die Einführung digitaler Technologien und Innovationen während der Krise auf Produktivität und Beschäftigung auswirken wird (Riom und Valero, 2020).

Mit den Investitionen in digitale Technologien ist ein Anstieg an IT-Schulungen einhergegangen. Auch für die nahe Zukunft sind in vielen Betrieben weitere Weiterbildungsaktivitäten geplant. Der Zusammenhang zwischen Investitionen und Schulungen zeigt sich vor allem bei den Betrieben, die pandemiebedingt in digitale Technologien investiert haben. In diesen Betrieben, die pandemiebedingt nachrüsten, fällt der Bedarf nach Weiterbildung im Bereich der Kommunikations- und Kooperationsfähigkeiten und des Datenschutzes besonders hoch aus.

Die Pandemie zwang die Firmen nicht nur, sich schnell auf eine Gesundheitskrise einzustellen; sie beschleunigte auch die Verbreitung und Nutzung digitaler Technologien, insbesondere in Kombination mit Homeoffice. Die Investitionen sind langlebig; es ist daher stark davon auszugehen, dass die damit verbundenen Veränderungen in den Arbeitsprozessen der Firmen und die flexiblere Gestaltung der Arbeit dauerhaft bleiben werden (Barrero et al., 2021).

## References

[CR1] Aminian, A. et al. (2021), Panel: Betriebe in der Covid-19 Krise — 20/21 Eine Längsschnittstudie in deutschen Betrieben — Welle 1–9, *FDZ-Datenreport*, 2.

[CR2] Barrero, J. M., N. Bloom, S. J. Davis (2021), Why Working from Home Will Stick, *NBER Working Paper*, 28731.

[CR3] Bellmann, L., P. Gleiser, C. Kagerl, E. Kleifgen, T. Koch, C. König, U. Leber, L. Pohlan, D. Roth, M. Schierholz, J. Stegmaier, A. Aminian, N. Backhaus und A. Tisch (2020), Potenzial für Homeofflce noch nicht ausgeschöpft, *IAB-Forum*, 21. Dezember.

[CR4] Bertscheck I (2020). Digitalisierung — der Corona-Impfstoff für die Wirtschaft. Wirtschaftsdienst.

[CR5] Janssen, S., U. Leber, M. Arntz, T. Gregory und U. Zierahn (2018), Mit Investitionen in die Digitalisierung steigt auch die Weiterbildung, *IAB-Kurzbericht*, 26.

[CR6] OECD (2021), OECD Employment Outlook 2021: Navigating the COVID-19 crisis and Recovery, OECD Publishing.

[CR7] Riom, C. und A. Valero (2020), The Business Response to Covid-19: The CEP-CBI Survey on Technology Adoption, *CEP Covid-19 Analysis*, 9.

[CR8] Taneja, S., P. Mizen und N. Bloom (2021), Working from home is revolutionising the UK labour market, VoxEU.org, 15. März.

[CR9] Wissenschaftlicher Beirat des BMWi (Bundesministeriums für Wirtschaft und Energie) (2021), Digitalisierung in Deutschland — Lehren aus der Corona-Krise.

